# Bioconversion of piceid to resveratrol by selected probiotic cell extracts

**DOI:** 10.1007/s00449-016-1662-1

**Published:** 2016-08-06

**Authors:** Mimoza Basholli-Salihu, Roswitha Schuster, Dafina Mulla, Werner Praznik, Helmut Viernstein, Monika Mueller

**Affiliations:** Department of Pharmaceutical Technology and Biopharmaceutics, University of Vienna, Althanstrasse 14, 1090 Vienna, Austria

**Keywords:** Probiotics, Piceid, Resveratrol, Bioconversion, Inflammation

## Abstract

Resveratrol exerts several pharmacological activities, including anti-cancer, anti-inflammatory, cardioprotective, or antioxidant effects. However, due to its occurrence in plants more in glycosidic form as piceid, the bioavailability and bioactivity are limited. The enzymatic potential of probiotics for the transformation of piceid to resveratrol was elucidated. Cell extract from *Bifidobacteria* (*B.*) *infantis, B. bifidum, Lactobacillus* (*L.*) *casei, L. plantarum,* and *L. acidophilus* was evaluated for their effect in this bioconversion using high-performance liquid chromatography (HPLC) as analytical tool. Cell extract of *B. infantis* showed the highest effect on the deglycosylation of piceid to resveratrol, already after 30 min. Cell extracts of all other tested strains showed a significant biotransformation with no further metabolization of resveratrol. The conversion of piceid to resveratrol is of importance to increase bioavailability and bioactivity as shown for anti-inflammation in this study. Cell extracts from probiotics, especially from *B. infantis,* may be added to piceid containing products, for achieving higher biological effects caused by the bioactivity of resveratrol or by health promoting of the probiotics. These findings open a new perspective of novel combination of cell extracts from probiotics and piceid, in health-promoting pharmaceutical and food products.

## Introduction

**Fig. 1:**
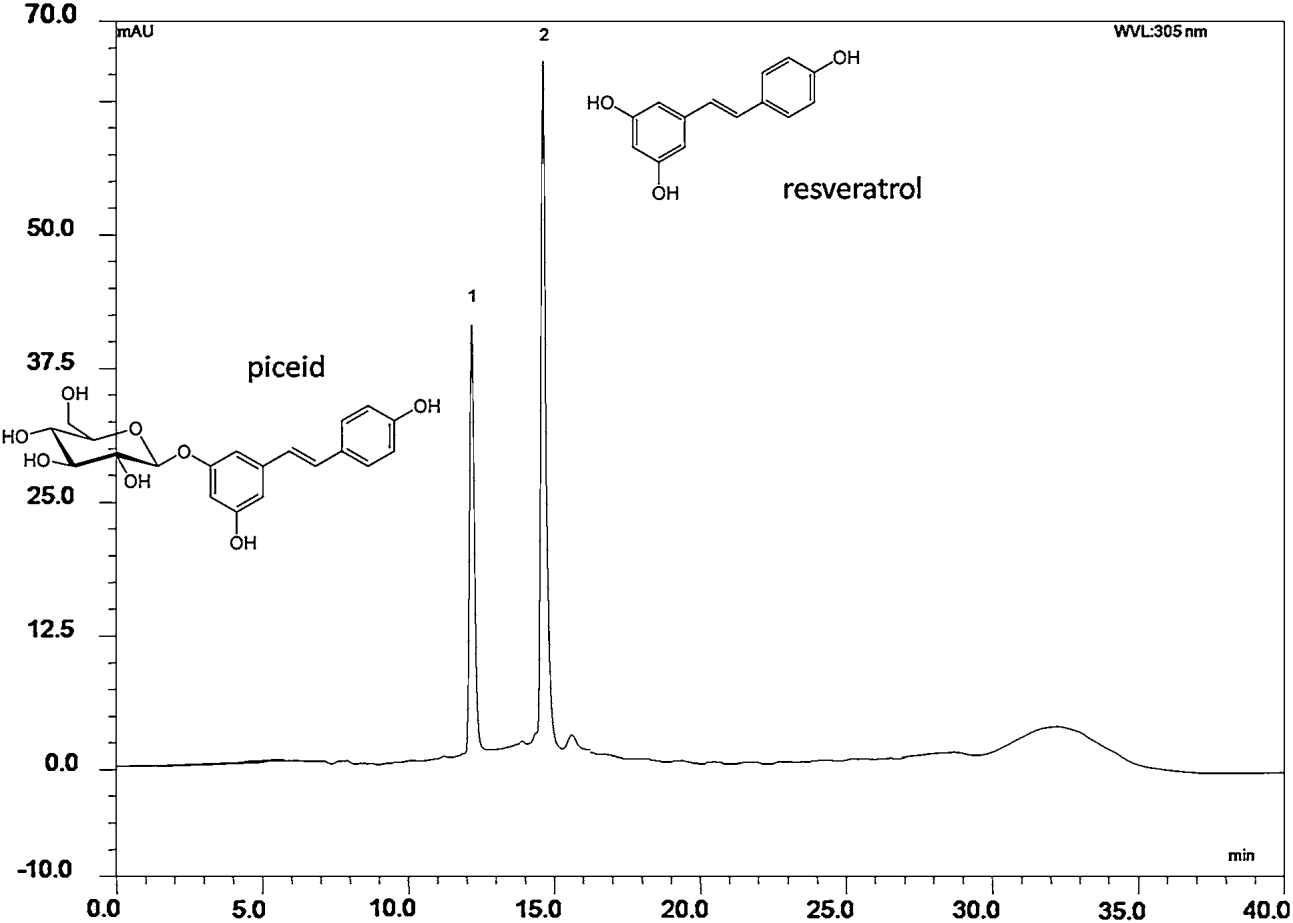
Structure and RP-HPLC chromatogram recorded at 305 nm showing piceid after incubation with enzymes of *L. plantarum* for 24 h at 37 °C; peak 1 (retention time of 12.1 min) corresponds to *trans*-piceid and peak 2 (retention time of 14.6 min) to *trans*-resveratrol

Resveratrol (3,5,4′-trihydroxystilbene) belongs to the group of plant polyphenols (stilbenes), found naturally in the grape skin and seeds, wine, berries, and medicinal plants, such as *Polygonum cuspidatum* [1, 2]. Several health benefits related to resveratrol, especially red-wine consumption, have been documented, such as protective effect against cancer, anti-inflammatory effect, cardiovascular protection, antioxidant activity, and inhibition of platelet aggregation [3–7]. *Trans*-resveratrol comes together with the less studied *cis*-resveratrol which occurs in a much smaller amount and has a similar biological effect [8–10]. In edible plants parts like grapes, including juices and wine, resveratrol occurs mainly in the glycosidic form, resveratrol-3-O-β-d glucoside, called piceid or polydatin (Fig. 1), which is present to a greater extent than resveratrol [10]. In grape juice, the concentration of piceid is around seven times higher than that of resveratrol and in cocoa, milk chocolate, and dark chocolate, the amount of piceid is four and five times higher than resveratrol, respectively [11]. Due to the lower bioactivity and bioavailability of piceid compared with resveratrol, the deglycosylation step is of high interest for developing bioactive products [12, 13].

The hydrolysis of piceid to resveratrol has been studied using the chemical techniques with acid and alkaline treatment [14] or by enzymatic conversion using crude fungal enzyme preparations [2, 15] or purified β-glucosidase from *Aspergillus oryzae* [2, 16], *Aspergillus niger*, yeast [17], or snailase [18]. Enzymes from probiotics, such as *Bifidobacterium* spp., showed the ability to deglycosylate isoflavone-glycosides, anthocyanin-glycoside, and quercetin-glucosides [19–21]. However, according to the best of our knowledge, no studies have been reported on the use of probiotics for the hydrolysis of piceid. The activity of hydrolases, such as glucosidase or rhamnosidase, is specific to the substrate, which means that the enzymes do not cleave the same sugar moiety from different aglycones to the same extent [22, 23]. Thus, the efficiency of the respective bioconversion has to be tested for each polyphenol-glucoside.

The bioconversion of piceid by probiotics would have several advantages, including receiving a safe product and a less expensive production compared with enzymatic (pure enzymes) and chemical hydrolysis. Furthermore, probiotics may colonize the gut microflora, thus providing a continuous system for deglycosylation in the human gut and exert additional health benefits [24].

The aim of this study was to evaluate the effect of five different probiotic cell extracts on the hydrolysis of *trans*-piceid to *trans*-resveratrol. The increase of bioactivity was confirmed in an anti-inflammatory in vivo test system.

## Materials and methods

### Chemicals, reagents, and bacterial strains

Piceid, resveratrol, Bradford reagent, p-nitrophenyl-β-d-glucopyranoside (p-NPG), and further chemicals were obtained from Sigma-Aldrich (St. Louis, MO, USA). *Bifidobacterium* (*B.*) *infantis* (UV16PR) was kindly provided by Medipharm (Kågeröd, Sweden), *B. bifidum* (BB12), *Lactobacillus* (*L.*) *acidophilus* (LA-5), and *L. plantarum* (ATCC 15697) were obtained from Christian Hansen A/S (Hørsholm, Denmark) and *Lactobacillus casei* 160 from Wiesby (Germany). Lyophylized cells of *Bifidobacterium* spp. were activated and grown in Reinforced Clostridial Medium (RCM, Oxoid Ltd., Hampshire, UK), while *Lactobacillus* spp. was activated and grown in De Man, Rogosa, and Sharpe broth (MRS, Oxoid Ltd., Hampshire, UK).

### β-Glucosidase activity assay

After the fourth subculture, cells were harvested by centrifugation (10,000×*g*, 4 °C, 10 min) and washed twice with 50 mM phosphate buffer pH 6.5. The cells were resuspended in the same buffer and mixed with glass beads (200–300 μm) to perform mechanical disruption of cells by mixing in five cycles, 2 min each with breaks on ice in between [25]. Then, the suspension was centrifuged (17,000×*g*, 30 min, 4 °C) to remove the cell debris and glass beads. The supernatant was then filtered through 0.45 μm filter (Merck Millipore, Darmstadt, Germany) and used for enzyme activity assays.

β-Glucosidase activity was determined according to the method described previously with minor modifications [25]. Briefly, 150 μL of 5 mM p-NPG in 50 mM phosphate buffer pH 6.5 were mixed with 600 μL of the appropriate dilutions of cell-free extract and incubated at 37 °C. The reaction was stopped on ice by adding 375 μL of cold 0.1 M NaOH. The amount of p-nitrophenol released from the reaction was measured at 410 nm with a Hitachi U-1100 spectrophotometer (Hitachi Ltd. Tokyo, Japan). The enzyme activity was expressed in Units (U), which defined the amount of p-nitrophenol released from the above reaction per ml per min (with a protein concentration of the cell extract of 1 mg/ml), under the reaction conditions. The protein content was determined using Bradford reagent [26] and bovine serum albumin (BSA) as standard. Optical density of the reaction mixture was read at 595 nm.

### Bioconversion of piceid by ß-glucosidase from probiotics

Bioconversion of piceid was carried out with cell extracts of *Bifidobacterium* spp. and *Lactobacillus* spp. Enzymatic hydrolysis of piceid was performed in triplicate by mixing 200 μL of 0.3 mM piceid in 50 mM phosphate buffer, pH 6.5, and 200 μL of cell-free extract. The stability of piceid was determined in the control samples prepared by mixing 200 μL of 0.3 mM piceid in 50 mM phosphate buffer pH 6.5 and 200 μL of 50 mM phosphate buffer pH 6.5. The negative control samples were prepared without piceid (200 μL of phosphate buffer pH 6.5 and 200 μL of cell-free enzyme). Reaction mixtures were incubated at 37 °C for up to 24 h. The bioconversion of piceid and, thus, the formation of resveratrol were determined after different incubation times, at the baseline (time 0 h), after 30 min, 2, 6, and 24 h. The reaction was stopped by adding methanol at the ratio 2:1 (v/v) to the aliquots. Then, the samples were centrifuged at 12,000*g* for 10 min (4 °C) and filtered through a 0.2 μm filter for the HPLC analysis.

### Analytical method for monitoring the bioconversion of piceid to resveratrol

Bioconversion of piceid to resveratrol was analyzed using high-performance liquid chromatography (HPLC) using a Dionex ICS 3000 HPLC, equipped with quaternary pump, autosampler, and PDA-100 diode array detector, and connected to Licrosphere RP C18 reverse-phase column (250 mm × 4.6 cm; particle size, 5 μm). Mobile phases used for analysis were 10 % (v/v) MeOH in water (solvent A) and 10 % (v/v) water in MeOH (solvent B), at a flow rate 0.5 ml/min, with the following gradient: 0 min: 100 % solvent A, 0–20 min: 0–90 % solvent B, 20–25 min: 90–60 % solvent B, 30 min: 60–0 % solvent B, followed by 10 min equilibrium period with initial conditions prior to the next sample injection (20 μL). Acquisitions of data were performed using the Chromeleon software (Dionex, Thermo Scientific, MA, USA) and a diode array detector set at wavelengths of 280 and 306 nm. The identified peak areas were compared with the calibration curve prepared from the standard solutions in methanol (0–250 μM for both standards). The calibration curves were prepared by plotting the concentration of standard solutions (μM) against the peak area. In this range of concentrations used, the calibration curves were linear with the regression coefficients *R* ^2^ = 0.998.

### Determination of anti-inflammatory activity

The potential anti-inflammatory effect was tested in murine macrophages (RAW 264.7, ATCC-TIB-71) stimulated with lipopolysaccharides (LPS). Cells were seeded at a density of 2 × 10^6^ cells per well in 12-well plates and incubated for 24 h at 37 °C. Cells were pretreated with test substances in DMSO (<0.1 %) for 3 h at 37 °C. Inflammation was stimulated by adding LPS at a final concentration of 1 µg/ml. After further 24 h of incubation at 37 °C, the cell supernatant was collected and centrifuged at 1500×*g* to remove cells.

The TNF-α, IL-6, and IL-10 secretion in the cell supernatants were analyzed using enzyme-linked immunosorbent assay (ELISA) assay according to the manufacturer´s protocol (Bioscience, San Diego, CA, USA) and using Infinite M200 microplate reader for measuring optical density (Tecan, Crailsheim, Germany).

In parallel, the viability of the cells was tested using a MTT (3-(4,5-dimethylthiazol-2-yl)-2,5-diphenyltetrazolium bromide) tetrazolium reduction assay. The cells were incubated in the presence of MTT for 2 h at 37 °C. The supernatant was then removed, and the cells were lysed with a buffer-containing 10 % SDS in 0.01 N HCl. The optical density at 570 nm, corrected by the reference wavelength 690 nm (to reduce the background), was determined.

The ELISA results were normalized to the MTT values to reduce any variation from differences in cell density. Concentrations with a viability reduction by 25 % or more would be excluded due to cytotoxicity. Cells treated with only LPS served as positive control and the resulting amount of secreted cytokines was defined as 100 %. The anti-inflammatory effect of dexamethasone, a known anti-inflammatory agent, was tested in parallel.

### Statistical analysis

All the experiments were done in triplicate in independent experiments on different days. Statistical analysis was performed using GraphPad Prism (GraphPad Software, San Diego, USA) using one-way ANOVA, and Tukey’s test cross comparing all study groups. Values of *p* < 0.05 were considered as significant.

## Results

### β-Glucosidase activity of *Bifidobacterium* spp. and *Lactobacillus* spp.

β-Glucosidase activity of the cell extracts of *Bifidobacterium* spp. and *Lactobacillus* spp. was determined using p-NPG as substrate and expressed in U/mg protein. As shown in Table 1, *B. infantis* cell extract significantly exerted the highest β-glucosidase activity among the probiotics tested (17.4 U/mg, *p* < 0.001). All other cell extracts showed a lower, but still significant β-glucosidase activity. No significant differences were observed of the enzyme activities between *L. acidophilus, L. casei,* and *L. plantarum,* namely 1.6, 1.5, and 1.3 U/mg, respectively (*p* > 0.05). *Bifidobacteria bifidum* cell extract has significantly shown the lowest β-glucosidase activity, namely 0.6 U/mg.

**Table 1:** β-Glucosidase activity of cell-free extracts of *Lactobacillus* spp. and *Bifidobacterium* spp.

	β-Glucosidase activity^a^
*B. infantis*	17.4 ± 0.5^b^
*B. bifidum*	0.6 ± 0.002^c^
*L. plantarum*	1.3 ± 0.008^d^
*L. casei*	1.5 ± 0.02^d^
*L. acidophilus*	1.6 ± 0.02^d^

^a^β-Glucosidase activity was expressed as units/mg protein. One unit was defined as the amount of p-nitrophenol released from p-NPG under the enzyme assay conditions, per ml per min

^b–d^Values (mean ± SD) with different superscript letters are significantly different (*p* < 0.05), *n* = 3

### Conversion of piceid to resveratrol by selected probiotic cell extracts

**Fig. 2:**
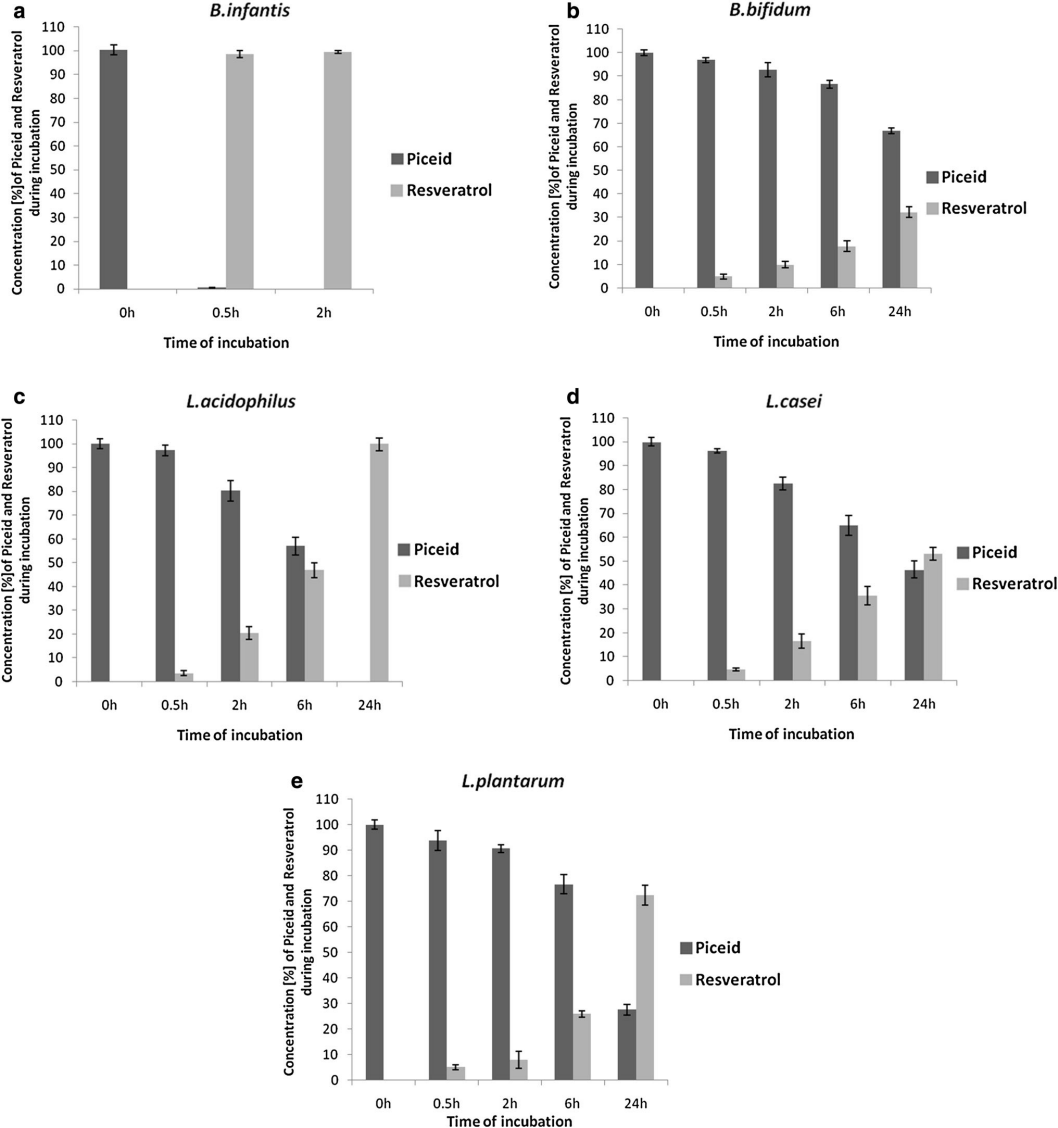
Enzymatic hydrolysis of piceid (*filled square*) and formation of resveratrol (*open square*) during incubation with cell-free extract of **a** *B. infantis*, **b** *B. bifidum* **c** *L. acidophilus,* **d** *L. casei,* and **e** *L. plantarum* at 37 °C. The values of piceid and resveratrol are expressed as percentage of the initial piceid concentration. During 24 h incubation of the control samples without cell extract of a probiotic, piceid remained stable

The ability of three cell extracts from *Lactobacillus* spp. and two strains of *Bifidobacterium* spp. on the transformation of piceid to its bioactive aglycone resveratrol was determined using HPLC (Fig. 1). All extracts significantly deglycosylated piceid. However, large differences in the yield and the rate of piceid bioconversion were observed. *B. infantis* showed the highest enzyme activity toward piceid, hydrolyzing 100 % of piceid to resveratrol within 30 min of incubation (Fig. 2a). *Lactobacillus acidophilus, L. plantarum,* and *L. casei* cell-free extracts showed lower conversion activity under the standard enzymatic conditions (Fig. 2). During the first 30 min of incubation, no significant differences were observed on the yield of conversion within the three strains of *Lactobacillus* spp. and *B. bifidum* (Fig. 2b). After 2 h of incubation, the yield of hydrolysis was 21 % for *L. acidophilus* (Fig. 2c). No significant differences were found for *L. casei,* showing a yield of hydrolysis of 16 % (*p* > 0.05) (Fig. 2d). After 2 h of incubation, *L. casei* and *L. acidophilus* showed significantly higher conversion yields in comparison with *L. plantarum* (8 % p < 0.01) (Fig. 2c–e). After 6 h of incubation, the rate of hydrolysis for *L. acidophilus* was 47 %, which was significantly higher (*p* < 0.001) than that observed for *L. casei* and *L. plantarum*, namely 36 and 26 %, respectively. After 24 h of incubation, piceid was completely converted to resveratrol in the presence of *L. acidophilus* enzymes. The rate of piceid hydrolysis by *L. casei* and *L. plantarum* was significantly lower, namely 53 and 72 %, respectively*. Bifidobacterium bifidum* has shown the lowest activity toward piceid among the probiotics tested in this study with 32 % conversion yield (Fig. 2b). During 24 h incubation of the control samples without cell extract of a probiotic, piceid remained stable. Furthermore, resveratrol was not further metabolized as observed at HPLC chromatograms (Fig. 1).

### Anti-inflammatory activity of resveratrol

**Fig. 3:**
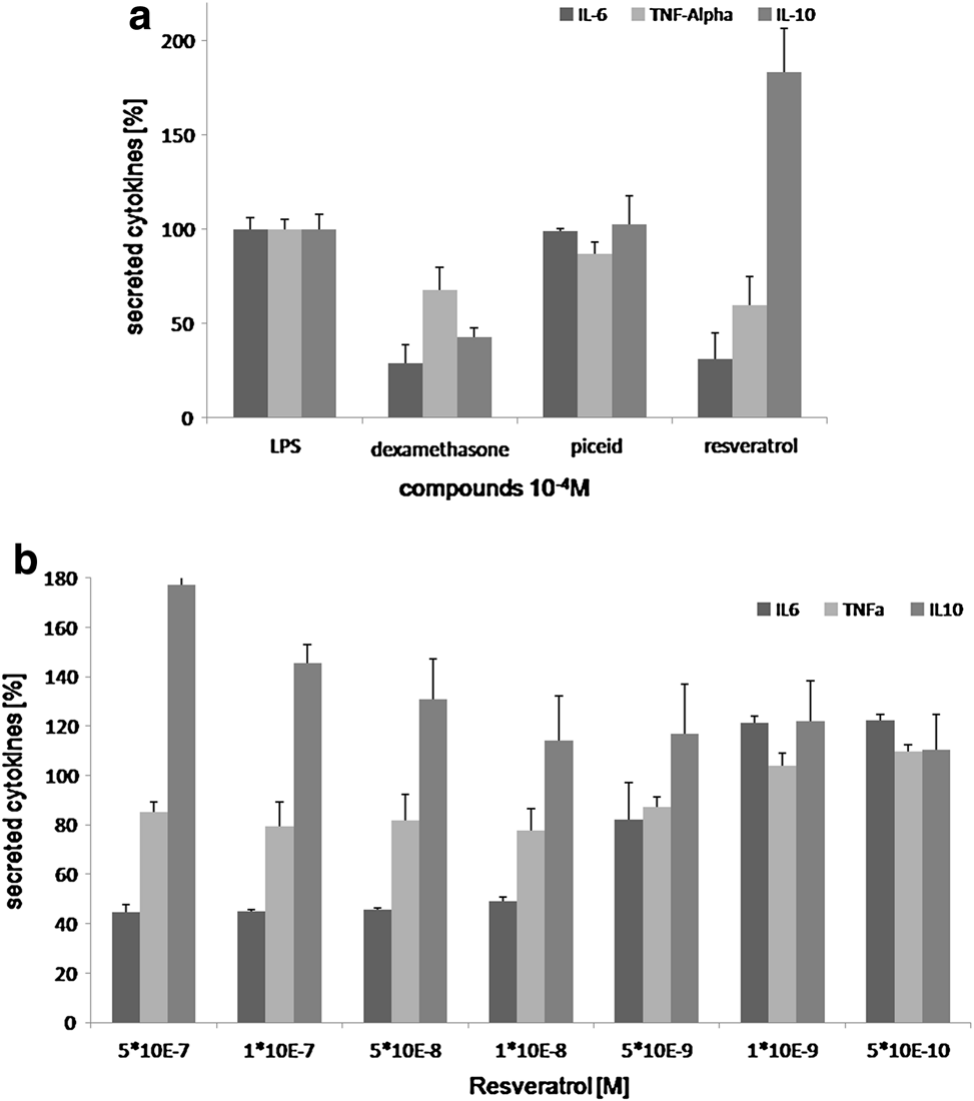
**a** Effect of resveratrol, piceid, and the positive control dexamethasone (10^−4^ M) on the cytokine secretion profile in LPS-stimulated macrophages. **b** Concentration-dependent effect of resveratrol (between 5 × 10^−7^ and 5 × 10^−10^ M) on the cytokine secretion profile. The concentration of cytokine secretion is expressed as percentage of the cytokine secretion with LPS only

Piceid showed no significant effect on the cytokine secretion, whereas deglycosylation to resveratrol led to a significant decrease of the secretion of the pro-inflammatory cytokines IL-6 and TNFα, and an increase of the anti-inflammatory cytokine IL-10 (Fig. 3a). This effect was dose-dependent (Fig. 3b).

## Discussion

The deglycosylation of piceid to resveratrol from plant sources is a crucial step to obtain sufficient bioavailability and bioactivity. As elucidated in this study, cell extracts of several probiotic strains, including *B. infantis, B. bifidum, L. acidophilus, L. casei,* and *L. plantarum* efficiently converted piceid to resveratrol. Among the probiotics tested, the highest β-glucosidase activity was observed for *B. infantis.* Differences in enzyme activity were shown for different probiotics strains previously [19, 21]. According to the best of our knowledge, this is the first study on the ability of piceid conversion by cell extracts from *Bifidobacterium* and *Lactobacillus* spp. However, the ability of *Bifidobacterium* spp. to convert isoflavone-glycosides and anthocyanine-glycoside was shown previously [19–21].

Since glycosidases are highly substrate specific, the efficiency has to be evaluated for each polyphenol-glycoside. In this study, no significant differences were observed in β-glucosidase activity toward the control substrate p-NPG among *Lactobacillus* spp. in contrast to the significant differences observed in piceid bioconversion. This is in agreement with the previous studies which showed no correlation between glucosidase activity and the yield of daidzein formation from its glycosidic form [27]. The probiotic β-glucosidase belongs to the group of glycosyl-hydrolases, which can hydrolyse the glycosidic bond between glucose moieties, present in gluco-oligosaccharides and glycosylated molecules, and can exert different specificities [28]. For the bifidobacteria tested, the glycolytic activity towards p-NPG correlated well with the effect on the piceid bioconversion. In such a way, the cell-free extract from *B. infantis* exerted the highest effect on p-NPG and also on piceid, whereas the cell-free extract from *B. bifidum* exerted the lowest effect on p-NPG and also on piceid.

It has to be considered that only the enzymatic activity of cell extracts was tested in this study. For using intact probiotics or for an effect in the human gut, piceid has to be diffusible through the cell membrane or the β-glucosidase has to be an exo-enzyme. This may be analyzed in further studies.

t-Resveratrol may be obtained from other sources as well, such as plant cell culture [29] or recombinant bacteria [30]. The level of resveratrol obtained from plant cell culture or the recombinant bacteria is three times higher than the level obtained with the lactobacilli. The level of resveratrol obtained with *B. infantis* is comparable with the level in the mentioned systems [29, 30].

For several diseases, it may be of importance to combine probiotics with anti-inflammatory agents to obtain a synergistic effect. The gut microflora of patients with inflammatory bowel diseases (IBD) or related diseases (for example) is changed to a lower number of *Bifidobacterium* and *Lactobacillus* [31] and thus to a lower ß-glucosidase activity by these strains. An alleviation of the symptoms of irritable bowel syndrome and inflammatory bowel diseases by probiotics could be shown [32, 33]. In addition, resveratrol has shown to improve the gut microflora balance through increasing the number of bifidobacteria and lactobacilli. However, there is a lack of studies on comparison of anti-inflammatory activities of piceid and resveratrol which would be a great potential for further investigations [34].

The results of this study lead to the suggestion that a combined formulation of piceid and probiotics would be advantageous. Piceid and probiotics may be incubated before consumption to start already hydrolyzing piceid and thus to obtain a more bioactive product. Furthermore, this conversion may be continued in the human gut. On the other hand, a colonization of the gut with these probiotic strains may provide the possibility of a continuous biotransformation in the human gut.

## Conclusion

Cell extracts of five different probiotic strains efficiently deglycosylated piceid within 24 h. *B. infantis* has shown the highest enzyme activity with complete bioconversion of piceid into resveratrol within 30 min. The β-glucosidase activity towards p-NPG cannot predict the activity towards piceid. This study confirms the substrate specificity of glycosyl-hydrolases from probiotics and, thus, the necessity for evaluation of each strain separately. These achievements open a new perspective for a combination of probiotics and piceid in food products for increasing the level of the health-promoting metabolite as resveratrol, prior or after consumption. Furthermore, the combination may be used as novel prodrug in pharmaceutical formulations. Synergic effects of the anti-inflammatory activity of resveratrol in combination with probiotics may be further evaluated as of major interest for diseases with chronic inflammation.

## References

[1] Burns J, Yokota T, Ashihara H, Lean M, Crozier A (2002). Plant foods and herbal sources of resveratrol. J Agric Food Chem.

[2] Zhang C, Li D, Yu H, Zhang B, Jin F (2007). Purification and characterization of piceid-β-d-glucosidase from *Aspergillus oryzae*. Process Biochem.

[3] Frémont L (2000). Biological effects of resveratrol. Life Sci.

[4] Damianaki A, Bakogeorgou E, Kampa M, Notas G, Hatzoglou A, Panagiotou S, Gemetzi C, Kouroumalis E, Martin P, Castanas E (2000). Potent inhibitory action of red wine polyphenols on human breast cancer cells. J Cell Biochem.

[5] Subbaramaiah K, Chung W, Michaluart P, Telang N, Tanabe T, Inoue H, Jang M, Pezzuto J, Dannenberg A (1998). Resveratrol inhibits cyclooxygenase-2 transcription and activity in phorbol ester-treated human mammary epithelial cells. J Biol Chem.

[6] Wenzel E, Soldo T, Erbersdobler H, Somoza V (2005). Bioactivity and metabolism of trans-resveratrol orally administered to Wistar rats. Mol Nutr Food Res.

[7] Mueller M, Hobiger S, Jungbauer A (2010). Anti-inflammatory activity of extracts from fruits, herbs and spices. Food Chem.

[8] Leiro J, Arranz JA, Fraiz N, Sanmartín ML, Quezada E, Orallo F (2005). Effect of cis-resveratrol on genes involved in nuclear factor kappa B signaling. Int Immunopharmacol.

[9] Orallo F (2006). Comparative studies of the antioxidant effects of cis- and trans-resveratrol. Curr Med Chem.

[10] Romero-Pérez A, Ibern-Gómez M, Lamuela-Raventós R, de La Torre-Boronat M (1999). Piceid, the major resveratrol derivative in grape juices. J Agric Food Chem.

[11] Hurst W, Glinski J, Miller K, Apgar J, Davey M, Stuart D (2008). Survey of the trans-resveratrol and trans-piceid content of cocoa-containing and chocolate products. J Agric Food Chem.

[12] Zhou J, Zhang H, Yang P, Li H (2002). Determination of resveratrol glucoside and resveratrol in radix and rhizome of *Polygonum**cuspidatum* yielded in Hanzhong Region. Chin Tradit Herb Drugs.

[13] Meng X, Maliakal P, Lu H, Lee M, Yang C (2004). Urinary and plasma levels of resveratrol and quercetin in humans, mice, and rats after ingestion of pure compounds and grape juice. J Agric Food Chem.

[14] Wang DG, Liu WY, Chen GT (2013). A simple method for the isolation and purification of resveratrol from *Polygonum cuspidatum*. J Pharm Anal.

[15] Chen M, Li D, Gao Z, Zhang C (2014). Enzymatic transformation of polydatin to resveratrol by piceid-β-d-glucosidase from *Aspergillus oryzae*. Bioprocess Biosyst Eng.

[16] Zhou L, Li S, Zhang T, Mu W, Jiang B (2015). Properties of a novel polydatin-β-d-glucosidase from *Aspergillus niger* SK34.002 and its application in enzymatic preparation of resveratrol. J Sci Food Agric.

[17] Jin S, Luo M, Wang W, Zhao C, Gu C, Li C, Zu Y, Fu Y, Guan Y (2013). Biotransformation of polydatin to resveratrol in *Polygonum cuspidatum* roots by highly immobilized edible *Aspergillus niger* and Yeast. Bioresour Technol.

[18] Wang Z, Zhao L, Li W, Zhang L, Zhang J, Liang J (2013). Highly efficient biotransformation of polydatin to resveratrol by snailase hydrolysis using response surface methodology optimization. Molecules.

[19] Tsangalis D, Ashton J, Mcgill A, Shah N (2006). Enzymic transformation of isoflavone phytoestrogens in soymilk by β-glucosidase-producing bifidobacteria. J Food Sci.

[20] Roncaglia L, Amaretti A, Raimondi S, Leonardi A, Rossi M (2011). Role of bifidobacteria in the activation of the lignan secoisolariciresinol diglucoside. Appl Microbiol Biotechnol.

[21] Otieno DO, Ashton JF, Shah NP (2006). Evaluation of enzymic potential for biotransformation of isoflavone phytoestrogen in soymilk by *Bifidobacterium* animalis, *Lactobacillus acidophilus* and *Lactobacillus casei*. Food Res Int.

[22] Amaretti A, Raimondi S, Leonardi A, Quartieri A, Rossi M (2015). Hydrolysis of the rutinose-conjugates flavonoids rutin and hesperidin by the gut microbiota and *bifidobacteria*. Nutrients.

[23] Ko J-A, Park JY, Kwon HJ, Ryu YB, Jeong HJ, Park SJ, Kim CY, Oh HM, Park CS, Lim YH, Kim D, Rho MC, Lee WS, Kim YM (2014). Purification and functional characterization of the first stilbene glucoside-specific β-glucosidase isolated from *Lactobacillus kimchi*. Enzyme and Microbial Technology.

[24] Nagpal R, Kumar A, Kumar M, Behare P, Jain S, Yadav H (2012). Probiotics, their health benefits and applications for developing healthier foods: a review. FEMS Microbiol Lett.

[25] Basholli-Salihu M, Mueller M, Salar-Behzadi S, Unger FM, Viernstein H (2014). Effect of lyoprotectants on β-glucosidase activity and viability of *Bifidobacterium infantis* after freeze-drying and storage in milk and low pH juices. LWT Food Sci Technol.

[26] Bradford MM (1976). A rapid and sensitive method for the quantitation of microgram quantities of protein utilizing the principle of protein-dye binding. Anal Biochem.

[27] Raimondi S, Roncaglia L, De Lucia M, Amaretti A, Leonardi A, Pagnoni U, Rossi M (2009). Bioconversion of soy isoflavones daidzin and daidzein by *Bifidobacterium* strains. Appl Microbiol Biotechnol.

[28] Schell M, Karmirantzou M, Snel B, Vilanova D, Berger B, Pessi G, Zwahlen M, Desiere F, Bork P, Delley M, Pridmore R, Arigoni F (2002). The genome sequence of *Bifidobacterium* longum reflects its adaptation to the human gastrointestinal tract. PNAS.

[29] Morales M, Bru R, Garcia-Carmona F, Barcelo AR, Pedreno MA (1998). Effect of dimethyl-b-cyclodextrins on resveratrol metabolism in Gamay grapevine cell cultures before and after inoculation with *Xylophilus ampelinus*. Plant Cell Tissue Org Cult.

[30] Watts KT, Lee PC, Schmidt-Dannert C (2006). Biosynthesis of plant-specific stilbene polyketides in metabolically engineered *Escherichia coli*. BMC Biotechnol.

[31] Neish AS (2009). Microbes in gastrointestinal health and disease. Gastroenterol.

[32] O’Mahony L, McCarthy J, Kelly P, Hurley G, Luo F, Chen K, O’Sullivan GC, Kiely B, Collins JK, Shanahan F, Quigley EMM (2005). *Lactobacillus* and *Bifidobacterium* in irritable bowel syndrome: symptom responses and relationship to cytokine profiles. Gastroenterology.

[33] Nobaek S, Johansson M-L, Molin G, Ahrné S, Jeppsson B (2000). Alteration of intestinal microflora is associated with reduction in abdominal bloating and pain in patients with irritable bowel syndrome. Am J Gastroenterol.

[34] Lanzilli G, Cottarelli A, Nicotera G, Guida S, Ravagnan G, Fuggetta M (2012). Anti-inflammatory effect of resveratrol and polydatin by in vitro IL-17 modulation. Inflammation.

